# A psychiatrist in training encounters a traditional healer

**DOI:** 10.4102/sajpsychiatry.v31i0.2453

**Published:** 2025-06-27

**Authors:** Raksha Singh, Pierre M. Joubert

**Affiliations:** 1 Department of Psychiatry, Faculty of Health Sciences, University of Pretoria, Pretoria, South Africa

**Keywords:** autoethnography, traditional healer, psychiatry training, shamanism, culture and psychiatry, ethnomedicine, indigenous medicine

## Abstract

**Background:**

Traditional healers play a significant role in healthcare seeking in South Africa. Many South Africans often seek healthcare services from both medical practitioners and traditional healers simultaneously for the same condition. Despite this, many medical practitioners seem ignorant about the practices of traditional healers.

**Aim:**

This study aimed to explore the similarities and differences between the practices of a traditional healer (TH) and a psychiatrist in training (PIT) regarding an inpatient mental healthcare user (MHCU).

**Setting:**

This study was conducted at an inpatient ward at Weskoppies Hospital, Pretoria.

**Methods:**

An autoethnographic method was utilised in this study.

**Results:**

The TH and PIT evaluated the same MHCU. While doing so, the PIT used participant observation, field notes, and finally a qualitative content analysis. The findings of the content analysis were validated with the TH. Two previously unpublished findings in South African traditional healing emerged: the use of a doll (effigy) and calling on angels.

**Conclusion:**

The TH and PIT followed the same basic steps in evaluating and treating the MHCU, but there were notable differences in the details (subcategories) of those steps. These differences reflect very different epistemologies about mental illness: the PIT used an evidence-based, naturalistic (or positivistic) model, while the TH used a model that can best be designated as transcendent.

**Contribution:**

This study contributes towards an understanding of a TH’s approach to a mentally disordered patient.

## Introduction

Traditional health practice, called ‘traditional medicine’ by the World Health Organization (WHO), is defined as:

[*H*]ealth practices, approaches, knowledge and beliefs incorporating plant, animal and mineral based medicines, spiritual therapies, manual techniques and exercises applied singular or in combination to treat, diagnose and prevent illnesses or maintain well-being.^1^

Related terms include indigenous healing,^2^ indigenous medicine,^3^ and ethnomedicine.^4^

Like most traditional forms of healing, South African traditional healing fits a shamanic approach to healing, but has been much less studied than other shamanisms.^5^ According to Matthews, shamanism is the oldest spiritual discipline in the world.^6^ Shamanism refers to a heterogeneous group of practices that has:

[…*I*]ts own universal symbolism and cosmology, inhabited by beings, gods and spiritual allies that show manifestly similar characteristics though they appear in localized forms depending on their place of origin.^6^

In Africa, ancestors play a prominent role, often acting as intermediaries between humans and God (in whichever way God is conceived).^5^

Traditional healing is not taken lightly in South Africa. The African National Congress Health Plan of 1994 stated that, ‘traditional healing will become an integral and recognised part of healthcare in South Africa’.^7^ It was also found that, ‘people have the right of access to traditional practitioners as part of their cultural heritage and belief system’.^7^ Traditional African health practices were formally recognised in South Africa with the promulgation of the *Traditional Health Practitioners Act, No.35 of 2004*,^8^ and later the *Traditional Healers Act, No 22 of 2007 (Act 22 of 2007)* provided a framework to ensure the efficiency, safety and quality of traditional healthcare services. This Act requires that traditional healers (THs) must be registered after training at an institution accredited by the relevant council set by the Department of Health.^9^

The African population of South Africa has a long tradition of medicinal use of indigenous plants to treat a variety of ailments, and many seek the advice of traditional healers.^10^ One study estimated that 8 out of 10 South Africans consult with a traditional healer,^11^ and in another research, it was observed that between 41% and 61% of South Africans suffering from a mental disorder consulted THs.^12^

Traditional healing needs to be researched and published in medical journals to inform medical practitioners about them.^13,14,15^ Through a better understanding of traditional healing practices, some medical practitioners were able to appreciate them.^16,17,18^ Nonetheless, many medical practitioners, being strongly committed to their own paradigm, view traditional healing negatively.^5^

South African studies on traditional healing targeted the physically ill,^9,19,20^ mentally ill^12,21,22^ and traditional healers as psychotherapists.^23,24,25^ Sorsdahl et al. used a qualitative method involving case vignettes to find out how THs regarded mental disorders.^22^ A study comparing the assessments of three THs with those of three psychologists found that psychologists and THs worked from different theoretical orientations, but there was a ‘significant degree of agreement’ regarding both diagnosis and treatment, implying a common perception of patients’ problems and needs.^18^ More recently, Koen et al. published an ongoing study among Xhosa schizophrenia sufferers recommending a more integrated approach between medical practice and traditional healing.^21^ No South African study on a psychiatric inpatient involving the direct collaboration between a psychiatrist in training (PIT) and a traditional healer ([TH] this abbreviation will only be used for the participant traditional healer) was found.

## About autoethnography

Autoethnography is situated in an ontology and epistemology of postmodernism, which holds that reality is known only by those who experience it personally and that reality is constructed through a process of self-conscious action.^26^ Autoethnography involves an in-depth study that uses an ideographic, qualitative approach.^27^ Autoethnography endeavours to describe and systematically analyse personal experience to understand cultural experiences.^28^ Narratives may be used to explore, describe, and offer a theoretical understanding of the issue under study.^26^ In this study, an autoethnographic approach with narratives between a participating patient and a PIT on the one hand, and a participating TH on the other hand, was used.

Usually, ethnographies are given in first person with extensive experiential descriptions, but limited space does not allow for it. Thus, descriptions must be brief while highlighting the most important findings.

## Aims and objectives

This study aimed to explore the similarities and differences between the practices of a TH and PIT in a mentally disordered inpatient to understand the way a specific TH evaluates and treats that patient.

### Methodology

#### Setting

The study was carried out at a specialist state psychiatric hospital (Weskoppies Hospital, Pretoria, South Africa).

#### Method

This study best fits ‘narrative autoethnography’,^28^ which emphasises the study of others by partly attending to encounters between the narrator and members of the group being studied.^28,29^ It uses interviews (patient and TH; patient and PIT; TH and PIT) and text (clinical notes, the TH’s notes and field notes) to accomplish its aims.^28^

Although autoethnography was used, I, the first author, while being a participant observer, did not (for practical reason) leave my own cultural setting (the hospital). On the contrary, the TH left his cultural setting to join me for the study in the hospital. Nonetheless, the essence of autoethnography was maintained by there being an interaction of two worlds.

The patient received standard, evidence-based psychiatric management while in hospital. She was not given any traditional medication and did not partake in any rituals during the study while in hospital. We evaluated the participant separately and did not discuss the participant until after the evaluation.

#### Traditional healer participant

The TH is registered with the Traditional Healer’s Council of South Africa. He completed his training to become a traditional healer after attending a school for traditional healing. He is in full time private practice. He informs that he can contact ancestors and multiple other spirits. Because he is Christian, he can also contact angels.

The first researcher visited two of his practices, but that is not where the encounter with the patient took place.

Patient participant: one patient was purposively selected as patient-participant for the study. She was a 35-year-old black, Sepedi speaking woman, who was admitted because of a disruptive, psychotic condition, characterised by hallucinations. She came from a family that held traditional beliefs. Her grandmother was a traditional healer. Her grandmother appeared in spirit to tell Mpho to become a traditional healer. After first ignoring the call, she said that she paid a price by experiencing life-problems. So, she went for training, completed it, but failed to fulfil a promise to do the final ceremony to honour her teacher and ancestors. The reason for this failure was a lack of money.

#### Data collection

I used written English to record field notes and the standard psychiatric interview, diagnosis and management. The TH used Sepedi for his notes. His wife translated the notes into English, and they were later also translated by the Department of African Languages, Faculty of Humanities, University of Pretoria to ensure accuracy.

#### Data analysis

A flexible method of qualitative analysis with thematic analysis as described by Braun and Clarke was used.^30^ Accordingly, first broad themes and then progressively finer themes within these were identified. Finally, the themes as emerging from the notes of TH and myself were compared. The themes were validated between myself, the TH and the second author.

### Ethical considerations

Ethical clearance to conduct this study was obtained from the University of Pretoria Faculty of Health Sciences Research Ethics Committee (No. 205/2014). The participants were able to provide written informed consent. Confidentiality is maintained by not revealing any demographic data that could identify the participant or traditional healer and by using pseudonyms for them.

## Results

I gave the participant the pseudonym, ‘Mpho’, and the TH, ‘Phenyo’.

Both of us spent about 90 min to evaluate Mpho. We did so separately. I was pleasantly surprised by how Phenyo, like myself, asked about Mpho’s auditory hallucinations in great detail. Phenyo found that hallucinations were of many human ‘voices’ and that Mpho could identify at least some of them. He found that ‘they’ were ‘speaking’ to one another and to her, and that she could differentiate between ancestors and other ‘voices’ (it was something she mentioned to me as well). He found that the ‘voices’ started after her training as a traditional healer and that before admission, the hallucinations intensified to the point of becoming disruptive. Phenyo had to dig deeper into the patient’s history, because at the time he evaluated her, the hallucinations were absent:

Phenyo: ‘How do you feel now?’

Mpho: ‘I feel well. Once I have taken the pills I usually sleep during the day. [*Also*], I don’t hear the voices anymore.’

Phenyo found the following during his history taking:

Phenyo: ‘Did you ever have a sudden shock or overwhelming fear? Also, when you hear these voices, do they seem like many voices?’

Mpho: ‘I couldn’t identify what these voices were saying. However, these voices did not scare me so much that I would run away. I have been hearing these voices for a time, so I have become used to them. I hear male and female voices. They do not sing; they just talk. They were becoming very noisy by the time I came to hospital.’

Phenyo: ‘Were you able to make out when the ancestors were talking to you as opposed to those other voices you mentioned?’

Mpho: ‘I could make out when the ancestors were talking, because they talked to me directly. I heard these [*problematic*] voices even after I completed my training in traditional healing. Before I went for the training there had been no [*problematic*] voices. When the ancestors visited me, they did not fight with those [*problematic*] voices. I tried to remove the [*problematic*] voices by traditional methods, but it failed. So, I am in hospital. The voices did not give me headaches – they were just voices. When I worked, I did not listen to them because I had already realised that they do not help me in any way. I was able to work even when I was hearing them. I [*also*] did not try sleeping pills to get rid of the voices, because I was sleeping well. The voices did not come at any specific time - they came at any time. [*Furthermore, they*] didn’t make me be impatient with my children and husband, but when I heard them. I did not interact with [*other*] people mainly because of them.’

Mpho told Phenyo that six ancestors talked to her and provided their names. On further questioning, she replied:

‘No, they only talk nicely, and they don’t fight’. (Mpho)

On questioning about whether they talked about the same topic, she answered:

‘Yes, when one talks, they all follow [with] the same [*topic*]’. (Mpho)

Both Phenyo and I recorded Mpho’s past psychiatry history. I noticed that Phenyo did not assume that it was Mpho’s first psychotic episode, because he also asked about previous such episodes. Both Phenyo and I asked about Mpho’s schooling while taking a past history. Phenyo also asked whether she had problems with headaches during school and whether she had difficulty reading the blackboard, which I did not do. When I asked him why this was important to him, he answered that such difficulties might indicate that black magic, intending to damage her mind, was inflicted on her at an early age.

Like me, Phenyo wanted to know about mental illness in the family. He clarified it by saying that such a history may indicate a genetic problem rather than a problem with the ancestors or black magic. Again, I was pleasantly surprised that the healer did not immediately make assumptions, but systematically excluded other possible (medical) causes for Mpho’s symptoms. He even considered genetic factors as a contributory factor to mental illness.

Like me, Phenyo enquired about Mpho’s childhood, abuse at home, and the quality of her marital relationship. He did so because, according to him, stress could damage the mind. He also asked about epilepsy, a possible head injury, accidents, and employment history.

Both of us also assessed the reliability of the information Mpho gave us. However, our means of seeking confirmation revealed significant differences in our epistemology; it will become clear when I describe how Phenyo and I managed Mpho. To assess Mpho’s reliability, I relied on collateral information from a social worker. Phenyo, on the other hand, relied on ancestors as well as on the patient’s uncle (a living relative). From this I inferred the following that Phenyo did not take everything the patient said for granted, and that he not only contacted the spirit world, but trusted the information he gathered by doing so.

Despite the similarities in the history taking, there were significant differences in recording the mental status examination. I recorded a detailed mental status examination in line with routine psychiatric practice. My own (extensive) records reflected that at the time I assessed her, Mpho displayed no psychopathology. Phenyo’s records were very succinct. That could be because he did not assess her any further because she said that she was feeling well. Thus, he might not have seen any reason for a more detailed assessment (this issue could not be clarified afterwards).

Once Phenyo and I had completed our initial assessments, we both took steps to finalise our diagnosis and treatment. We did so in very different ways. I prepared my patient and clinical notes for presentation to a multidisciplinary team (MDT), as is standard practice at the hospital. This MDT consisted of a psychiatrist, clinical psychologist, social worker, occupational therapist and a professional nurse. A diagnosis of brief psychotic disorder was made according to the DSM-IV-TR criteria.^31^ The MDT decided on psychotherapy, occupational therapy, and an antipsychotic drug, which was to be tapered down in due course under proper circumstance. Phenyo, however, followed a completely different course of action.

Once Phenyo had completed his assessment, he made no preliminary diagnosis. He first sought clarification about the diagnosis and treatment from the spirit world. Accordingly, he went to his practice, where he fasted, prayed, and entered an alternate state of consciousness (also called a ‘non-ordinary reality’^5^). Firstly, he fasted for three days; then he prayed to angels. Secondly, he contacted Mpho’s ancestors. Thirdly, he contacted his own ancestors and, finally, he contacted Mpho’s ancestors again. It will soon become clear why these steps were taken – it was certainly a revelation to me. I did not expect angels to come into the picture. Phenyo told me that it was because of his Christian background and that he believed in both Christianity and the ancestors. Thus, his practice integrates Christian beliefs with shamanistic beliefs. I also did not expect a to-and-fro contact between Phenyo and two different ancestor groups. His reason for his having done so, will soon become evident.

When Phenyo contacted the angles, he wanted to know the following: Was Mpho bewitched? Is she a traditional healer, and if so, did she cure a bewitched person? Who were Mpho’s protective angels and where did they stand? Could she be healed of all her problems? Also, ‘What medication should we take spiritually so that she can be healed?’

Twenty minutes later, he received answers from the angels. He told me that they communicated with him in his mind. The answers he received were that Mpho was not bewitched, that she did not heal someone who was bewitched, and that:

‘The angels are there for her, but they are disappointed [*in her*]. They said that she will never get healed by spiritual practices [*meaning Christian interventions*], but only by traditional practices. Thereafter they kept quiet.’ (Phenyo)

Phenyo reported that Mpho had many ancestors who made her ill because all of them wanted to work with her. Consequently, all of them were talking at the same time when she approached them. He said her ancestors did it to him too, so much so that he did not get a chance to speak. That is the reason why he stopped talking to them and, instead, contacted his own ancestors. He asked his own ancestors how he could get the cooperation of Mpho’s ancestors. His ancestors told him to burn two herbs (*hlahlabadimo* and *kobamadhaze*) over hot coals. While doing so, Phenyo had to introduce himself to Mpho’s ancestors while adding another herb [*mphepe*], which would calm her ancestors and allow him to control [*sic*] them. Phenyo also had to put snuff on the ground to create a boundary within which he could contain and control Mpho’s ancestors. In doing so, he would get only the most powerful ancestors to talk to him. The ancestors contacted him after an hour and asked who he was. He introduced himself as *Kobela* [a senior person] and a traditional healer who trains other traditional healers. At this, her ancestors were satisfied and asked what he wanted.

Phenyo established from Mpho’s ancestors that, if she did as they desired, they would leave (her in peace). Their grievance was that she had separated out her ancestors into maternal and paternal lines. But, although traditional healing comes from the maternal line, her paternal ancestors also demanded recognition. Mpho’s ancestors were very angry at her because she had not completed her training in order to bring back their *thebele* [property – this included what the ancestors gave her to use in her treatment of people]. Phenyo then tried to appease them, but without success. However, they provided him with a list of ritual tasks for Mpho to complete to come into consideration for forgiveness.

Phenyo enquired into Mpho’s TH training in detail such as: Who had taught her? Was she able to use *mmankgonyane*. Phenyo said that *Mmankgonyane* is a doll used by ancestors that is protected by a secret ancestral medicinal mixture [*pheko*], which enables the doll to identify all illnesses in a person as well as the medicines for healing, Was she able to use *ditaola* (according to Phenyo, diagnostic bones used by ancestors to diagnose peoples’ illnesses and to identify medicines for treatment)? Had she healed people? Did she know the medicines used by traditional healers? I was excited to discover that, contrary to popular belief, this TH does not only use bones for diagnosis, but also a doll [*effigy*]. I felt privileged to be allowed access into the TH’s world.

Mpho’s ancestors said that if things did not go well, Mpho must go to a river to say goodbye to the ancestors without fear. The ancestors will then take back their gifts. When she comes back, she must bathe in noisy water (water from a stream) combined with milk so that the ancestors can see to whom they will bestow their gifts in place of Mpho. They threatened to make Mpho blind if she should try to regain the gifts after this, but otherwise they leave her in peace.

I found this entire process of contacting the spirit world fascinating and worlds apart from my medical training. The healer showed respect towards the ancestors. He did not make demands on them, but rather tried to collaborate with them in a type of team effort. Phenyo told me that if he should find a patient in his practice that he deems mentally ill, and if this is also confirmed by the patient’s ancestors, he would refer that patient to the nearest hospital using his standard traditional healer referral of letter.

## Discussion

There were both similarities and differences in the process of evaluation and treatment between the TH and me. Some of the steps were similar, namely: demographic information, presenting complaint, further inquiry into symptoms and past history, as well as other investigations and management. The time spent in consultation was similar (about 90 min). However, in line with Edward, and not surprisingly, there were differences in the theoretical approach.^18^ A physical examination on the patient was performed, and I took detailed notes on history and mental status examination in order to present her to an MDT for final diagnosis and evidence-based management.

The TH did not do a physical examination. This is common among traditional healers, who would rather rely for revelations from the spiritual world.^5^ Also, the TH’s notes were not as detailed. His notes reflected an intention to rule out mental illness, medical illness and substances. If those are ruled out, the patient’s problems might be considered as of a spiritual nature. For the spiritual nature of her disorder, the TH took matters up with the spiritual world.

The foregoing indicates two very different theoretical paradigms: the naturalistic or materialistic paradigm of a PIT, and the transcendental paradigm of the TH. ‘Transcend’ as used here means going (seemingly) beyond oneself.^32^ The paradigm I (the PIT) used is well described and needs no elaboration. The transcendental paradigm of TH will be discussed. This paradigm is embedded in African religion, which is diverse and complex but has commonalities.^33^

Two previously unpublished phenomena emerged from this study: angels and a doll (as a type of effigy). How common the use of effigies and angels are in South African traditional medicine, has, to our knowledge, not been published. However, the use of divination is common in African traditions. Effigies are used for divination.^34^ Furthermore, if an effigy and angels are employed by one TH as in this study, there are likely to be a wider use of them. For example, Turner described the use of effigies for divinations by the Ndembu of Zambia.^35^ The Ndembu used divination for community healing.^35^ Ancestral spirits are a known part of South African traditional healing.^2,5^ Edwards found a Christian style faith healing appearing in local traditional healing with, for example, the advent of the *umthandazi* or *umpropheti* (faith healers in the Christian tradition) by 1986.^18^ If Phenyo, who described himself as a Christian, consults angels, one would expect that there are very likely other traditional healers who do the same. After all, African traditional healers also follow a Christian religion as can be inferred from the following quote:

Today, many [*traditional healers*] are active members in major religions, mainly Christian religions. … [*Traditional healers*] recognise that Traditional Healing and Traditional Medicine go hand in hand with Christian and other religious beliefs.^36^

Furthermore, praying to angels has been popularised by people such as Doreen Virtue.^37^ The use of a doll (or effigies) was not mentioned by Cumes or Edwards.^2,18^ Apart from angels and the doll, the TH used many of the methods described by Cumes^2^: he contacted ancestors, used bones, snuff, different herbs, and prescribed rituals.

The TH established the cause of the patient’s disorder as her aggrieved ancestral spirits, because she did not complete her training as a traditional healer and neglected to appreciate her paternal ancestral line. Cumes mentioned that ancestral spirits may, on rare occasions, cause illness.^2^

Having discussed the findings, I will share my thoughts about this research experience. I can only do so from my own background, namely that of a practising Hindu following the Vedic scriptures, who was also trained in a scientific approach to medicine. That having been said, my parents brought me up to be open-minded, and to seek my own truths regarding religion, spirituality, and many other things, while at the same time respecting other cultures and religions.

Embarking on this research was a learning adventure for me, which I hope to take further at a later stage. I embarked on the study trying to be as objective as possible in order to learn from it. I have learned how the TH managed the patient, and I guess he will follow much the same procedures with his other patients, but I also found that I have questions that I could not answer. Did he really contact the spirits of ancestors? Did he really contact an angel? Were his experiences at some level objectively real, or was it dissociative imaginings? Would the interventions, if implemented, contribute to the patient’s mental well-being? I know that there are many unexplained phenomena in the world and shall accept that I have more questions than answers. I shall keep an open mind and maybe I shall find more certainty in future.

I guess that many medical practitioners will think the same way I do, but that some will immediately consider shamanic practices nonsense. I can understand the latter stance, because I realise that the paradigms from which we medical practitioners work are very different from those of shamans. That having been said and my doubts having been aired, I am nonetheless of the opinion that THs are here to stay and that they can meaningfully contribute to the well-being of our patients, at least in some cases. I think that such contribution could be especially useful in developing countries with limited resources. At the same time, I realise that THs may, at times, harm patients, but that goes for medical practitioners too.

## Limitations

The findings of the study are clearly limited to what was learned by a single interaction between TH and PIT around one patient. As all autoethnographic work, it is meant to report one researcher’s experience and thoughts and thus, is to report on a representative sample. Furthermore, in hindsight, much more could have been learned about the detailed involvement of a doll and angels. That being so, the study indicates that cooperation between a TH and PIT is possible, while each hold to their own expertise. Furthermore, the findings are in line with a previous study.^18^ The patient’s experience and perspective were not included in the study, but, in hindsight, it would have been valuable.

## Recommendations

This study and previous studies indicate that collaboration between THs and medical practitioners is possible. It could be argued that in a country with such a diverse population, it is necessary. But, before such collaboration could be implemented, more studies, initially probably qualitative, but later perhaps quantitative, are recommended. It would be valuable to add patients’ perspectives.

## Conclusion

In this autoethnography, a TH and a PIT followed the same basic steps in evaluating and treating a mentally disordered patient. However, there were notable differences in the epistemologies about mental illness: the PIT using an evidence-based psychiatric paradigm, while the TH used what is best labelled a transcendental model. Two previously unpublished phenomena in South African traditional healing emerged: the use of angels and a doll (effigy).
